# Impact of caesarean section on mode of delivery, pregnancy-induced and pregnancy-associated disorders, and complications in the subsequent pregnancy in Germany

**DOI:** 10.3205/000233

**Published:** 2016-06-14

**Authors:** Louis Jacob, Sevil Taskan, George Macharey, Ingeborg Sechet, Volker Ziller, Karel Kostev

**Affiliations:** 1 Department of Biology, École Normale Supérieure de Lyon, Lyon, France; 2 Department of Gynecological Endocrinology and Reproductive Medicine, Hospital of Gynecology and Obstetrics University, Hospital Gießen und Marburg, Marburg (Lahn), Germany; 3 Department of Obstetrics and Gynecology, Helsinki University and Helsinki University Central Hospital, Helsinki, Finland; 4 IMS Health, Frankfurt, Germany

**Keywords:** caesarean section, mode of delivery, pregnancy-induced disorders, pregnancy-associated disorders, pregnancy

## Abstract

**Objectives:** To analyze the impact of caesarean section (CS) on mode of delivery, pregnancy-induced and pregnancy-associated disorders, as well as complications in the subsequent pregnancy within German gynecological practices.

**Methods:** 1,801 women with CS and 1,801 matched women with vaginal delivery (VD) from the IMS Disease Analyzer database were included. The impact of previous CS on the mode of delivery and pregnancy-associated disorders as well as complications prior to or during birth in the subsequent pregnancy were analyzed. Cox regressions were used to determine the influence of CS with regard to these outcomes.

**Results:** Medical abortion and single spontaneous delivery were significantly less frequent in women with a history of CS compared to VD (OR equal to 0.52 and 0.04 respectively), whereas CS after CS was the significantly more common mode of delivery (79.0% versus 9.3%, OR=36.47). Gestational hypertension without significant proteinuria, gestational hypertension with significant proteinuria, and polyhydramnios were more frequent in women with CS than in women with VD (OR equal to 6.80, 1.71, and 2.29). Hemorrhage and maternal care for known or suspected disproportion were more common in the CS group than in the VD group (OR equal to 1.34 and 3.75). Prolonged pregnancy, preterm labor, abnormalities arising from forces of labor, and perineal laceration during delivery were significantly less frequent in women with CS than in women with VD (OR between 0.32 and 0.75), whereas long labor was more common (OR=2.09).

**Conclusion:** Women with CS were more likely to undergo further CS and to develop major pregnancy-associated diseases in the following pregnancy compared to women with VD.

## Zusammenfassung

**Ziele:** Analyse der Auswirkungen eines Kaiserschnitts (KS) auf die Art der Entbindung, schwangerschaftsinduzierte und schwangerschaftsassoziierte Erkrankungen sowie auf Komplikationen bei der anschließenden Schwangerschaft in deutschen gynäkologischen Praxen.

**Methoden:** Insgesamt wurden 1.801 Frauen mit KS und 1.801 Kontrollpatientinnen mit Vaginalgeburt (VG) aus der IMS Disease Analyzer-Datenbank in die Studie eingeschlossen. Die Auswirkungen des vorherigen KS auf die Art der Entbindung und auf schwangerschaftsassoziierte Erkrankungen sowie auf Komplikationen vor oder während der zweiten Geburt wurden analysiert. Cox-Regressionsanalysen wurden verwendet, um den Einfluss eines KS in Bezug auf diese Auswirkungen zu bestimmen.

**Ergebnisse:** Medizinische Abtreibungen und spontane Einzelgeburten kamen bei Frauen mit einem KS in ihrer Vorgeschichte signifikant seltener vor als bei Frauen mit VG (OR=0,52 bzw. 0,04), während KS nach KS die signifikant häufigere Art der Entbindung war (79,0% verglichen mit 9,3%, OR=36,47). Schwangerschaftshypertonie ohne signifikante Proteinurie, Schwangerschaftshypertonie mit signifikanter Proteinurie und Polyhydramnie waren bei Frauen mit KS häufiger als bei Frauen mit VG (OR=6,80, 1,71 und 2,29). Blutungen sowie Behandlungen während der Schwangerschaft im Hinblick auf bekannte oder vermutete Disproportionen waren in der KS-Gruppe häufiger als in der VG-Gruppe (OR=1,34 bzw. 3,75). Übermäßig lange Schwangerschaften, vorzeitige Wehen, durch den Geburtsprozess verursachte Anomalien und Dammrisse während der Entbindung waren bei Frauen mit KS signifikant seltener als bei Frauen mit VG (OR zwischen 0,32 und 0,75), wohingegen lange Geburten häufiger auftraten (OR=2,09).

**Schlussfolgerung:** Frauen mit KS unterziehen sich eher einem weiteren KS und leiden in der folgenden Schwangerschaft häufiger an schweren schwangerschaftsassoziierten Erkrankungen als Frauen mit VG.

## Introduction

Caesarean section (CS), also known as C-section, is a surgical procedure which has been increasingly used in the past decades [1], [2], [3]. Approximately 18.5 million CS are performed each year worldwide [4]. In 2014, 31.8% of pregnant women give birth by CS in Germany, emphasizing the importance of this surgery in this country [4]. 

CS has been at the center of an intensive debate in recent years. Some authors have suggested that CS may be deleterious to both mother and baby [5], [6]. In 2003, Smith and colleagues found that the rate of antepartum stillbirths was significantly higher in the CS group compared to the VD group [5]. However, their study also included births of twins. Later, in 2006, Silver et al. demonstrated that the risks of placenta accreta, cystotomy and other complications increased with a rising number of CS deliveries [6]. Moreover, further studies could show that children delivered by CS had significantly higher risk for type 1 diabetes [7], autism spectrum disorder [8], asthma [9] and obesity [10] than children born by VD. By contrast, a retrospective study has shown that CS has little or no effect on future fertility [11]. Therefore, the debate concerning the risk of CS on future pregnancies remains current.

Although Germany has a high CS rate, there is a lack of comprehensive epidemiological studies on the effect of CS on the mother and the baby in this country. Thus, our goal was to analyze the impact of CS on mode of delivery, pregnancy-induced and pregnancy-associated disorders, and complications arising during the subsequent pregnancy in Germany. 

## Methods

### Database

The Disease Analyzer database (IMS Health) compiles drug prescriptions, diagnoses, basic medical and demographic data obtained directly and in anonymous format from computer systems used in the practices of gynecological practitioners [12]. Diagnoses (ICD-10), prescriptions (Anatomical Therapeutic Chemical (ATC) Classification System) and the quality of reported data have been monitored by IMS based on a number of criteria (e.g., completeness of documentation, linkage between diagnoses and prescriptions). 

In Germany, the sampling methods used for the selection of physicians’ practices were consistent with a representative database of gynecological practices [12]. Prescription statistics for several drugs were very similar to data available from pharmaceutical prescription reports [12]. The age groups for given diagnoses in Disease Analyzer were also commensurate with those in corresponding disease registries [12]. 

### Study population 

Between January 2000 and December 2013, 10,195 women gave birth by CS and 16,132 gave birth by VD for the first time in the German gynecological practices identified in the IMS Health database. We selected women who were pregnant a second time. After matching (1:1) women with CS and women with VD using age and diagnosis of obesity (ICD 10: E66) as criteria, 1,801 subjects were included in each group (see data publication [13]).

### Study outcome

The primary outcome measure was the impact of CS on the mode of delivery (CS or VD) in subsequent pregnancy. The impact of CS on the most common pregnancy-induced diseases, pregnancy-associated disorders, and complications prior to or during birth was also analyzed. 

### Statistical analysis 

Cox regressions were used to determine the influence of CS with regard to the mode of delivery, pregnancy-induced diseases, pregnancy-associated disorders, and complications prior to or during birth in subsequent pregnancy. A p-value <0.05 was considered statistically significant. All calculations were carried out using SAS 9.3 (SAS Institute, Cary, USA).

## Results

### Impact of caesarean section on mode of delivery in subsequent pregnancy

Table 1 (Tab. 1) displays mode of delivery in women with prior CS or VD in German gynecological practices. The study included 1,801 women with CS and 1,801 women with VD. Medical abortion and single spontaneous delivery were significantly less frequent in women with CS than in women with VD (OR=0.52, 95% CI: 0.35–0.78; and OR=0.04, 95% CI: 0.03–0.05), whereas CS was more common (79.0% versus 9.3%, OR=36.47, 95% CI: 30.01–44.33). Unspecified abortion and single delivery by forceps and vacuum extractor did not differ between the two groups (p-values equal to 0.1979 and 0.4334 respectively). 

### Impact of caesarean section on pregnancy-induced and pregnancy-associated diseases, and on complications prior to and during birth

Table 2 (Tab. 2) illustrates pregnancy-induced maternal disorders in women with CS and women with VD. Gestational hypertension without significant proteinuria, gestational hypertension with significant proteinuria, and polyhydramnios were more frequent in women with CS than in women with VD (OR=6.80, 95% CI: 3.22–14.33; OR=1.71, 95% CI: 1.19–2.47; and OR=2.29, 95% CI: 1.33–3.95). The frequencies of gestational oedema and proteinuria without hypertension, eclampsia, placenta disorders, and placenta previa were not significantly different between the two groups. Table 3 (Tab. 3) displays pregnancy-associated disorders in women with CS and women with VD. Hemorrhage and maternal care for known or suspected disproportion were more common in the CS group than in the VD group (OR=1.34, 95% CI: 1.12–1.61; and OR=3.75, 95% CI: 2.45–5.68). Contrastingly, maternal care for known or suspected malpresentation and antepartum hemorrhage did not significantly differ between women with CS and women with VD. Finally, Table 4 (Tab. 4) shows complications prior to and during birth in women with prior CS or VD. Prolonged pregnancy, preterm labor, abnormalities of forces of labor, and perineal laceration during delivery were significantly less frequent in women with CS than in women with VD (OR between 0.32 and 0.75), whereas long labor was more common (OR=2.09, 95% CI: 1.05–4.19). Rates of premature rupture of membranes, premature separation of placenta, false labor, obstructed labor due to malposition and malpresentation of the fetus, other obstetric trauma, postpartum hemorrhage, retained placenta and membranes without hemorrhage, and other complications of labor and delivery did not significantly differ in our study. 

## Discussion

In this German retrospective study, it was shown that women with CS were less likely to have a single spontaneous delivery and more likely to undergo CS in a subsequent pregnancy compared to women with VD. CS was also associated with a higher risk for certain outcomes such as gestational hypertension, polyhydramnios, hemorrhage in early pregnancy, maternal care for known or suspected disproportion and long labor in subsequent pregnancy. Contrarily, previous CS was associated with a lower risk of medical abortion, prolonged pregnancy, preterm labor, abnormal forces of labor, and perineal laceration during delivery in the next pregnancy compared to VD. 

Although there is a significant number of articles on the benefits and the risks of CS, there is a lack of comprehensive epidemiological studies on the effects of this surgical procedure in Germany. Therefore, new analyses with recent data from the IMS Health database were performed. The first major outcome of this work revealed that previous CS is associated with a 36-fold increase in the odds of CS in subsequent pregnancy. Though several authors have studied the impact of repeated CS on both mother and baby [6], [14], [15]. There is still little information on the actual effect of prior CS on subsequent CS [16], [17], [18]. The findings from this study are not surprising from an obstetrical point of view. For example, it is recognized that after CS, the next child has a 70% chance of likewise being delivered by CS ([19], p. 34). 

Indeed, although women believe that VD is more natural than CS, it is associated with considerable side effects (i.e. urinary incontinence and neuropathy) [20], [21]. In a study comprising 15,307 women in 2003, Rortveit and colleagues found that VD significantly increased the risk of urinary incontinency compared to CS (OR=1.7, 95% CI: 1.3–2.1) [21].

Moreover, it was shown that CS was associated with a reduction in the rates of medical abortion, prolonged pregnancy, preterm labor, abnormalities of forces of labor, and perineal laceration during delivery. 

Nonetheless, another important result of this work is that CS is associated with elevated risk of several adverse outcomes involving short- and long-term effects on both the mother and the baby. It was discovered that CS was associated with a 7-fold increase in the risk of developing gestational hypertension without significant proteinuria as well as with a 2-fold increase in the probability of developing gestational hypertension with significant proteinuria. It was shown that CS had a major impact on polyhydramnios (OR=2.29, 95% CI: 1.33–3.95). These findings correspond with the existing literature, as several studies have already demonstrated that CS has a negative effect on morbidity and mortality. In a study including 120,633 singleton second births, Smith and colleagues have found that the odds of unexplained stillbirth was significantly higher with prior CS than with prior VD (HR=2.74, 95% CI: 1.74–4.30). In 2006, Silver et al. further showed in 30,132 women examined between 1999 and 2002 in 19 American academic medical centers that the risk for placenta accreta increased with the number of CS (≥6: OR=15.2, 95% CI: 6.9–33.5, when compared with one CS) [6]. Thus, even when the use of CS might have a positive impact on the health of both mother and the baby, it is associated with major side effects, which emphasizes the importance of continuous reevaluation of the CS-related risk-benefit balance. 

Retrospective primary care database analyses are generally limited by the validity and completeness of the data on which they are based. The present study includes several limitations, such as the assessment of abortion and the definition of CS use and co-morbidities, which relied solely on ICD codes entered by gynecologists in gynecological practices, not by the obstetrician. Another important limitation of this work is that only two variables (age and diagnosis of obesity) were used for matching women with CS and women with VD, resulting in potential bias factors. Furthermore, age, and data pertaining to socioeconomic status (e.g., education, income) and lifestyle-related risk factors (e.g., smoking, alcohol, physical activity), were also lacking due to legal restrictions for data privacy protection. Finally, Disease Analyzer database did not allow us to separate elective Caesarean for breech, Caesarean for other indications, and emergency Caesarean.

Overall, the present study indicates that women with CS in German gynecological practices were less likely to have a single spontaneous delivery and more likely to undergo CS in a subsequent pregnancy compared to women with VD. Interestingly, CS was associated with both the increase and decrease of the risk of major diseases and disorders associated with pregnancy. Congruent with the literature, these data underscore the need for intensive management and follow-up of women with CS. Furthermore, new studies are required to reevaluate the CS-related risk-benefit balance, and to gain a better understanding of the association between prior and subsequent CS. 

## Data

Data for this article are available from the Dryad Repository: http://dx.doi.org/10.5061/dryad.g7t04 [13].

## Notes

### Competing interests

The authors declare that they have no competing interests.

## Figures and Tables

**Table 1:**
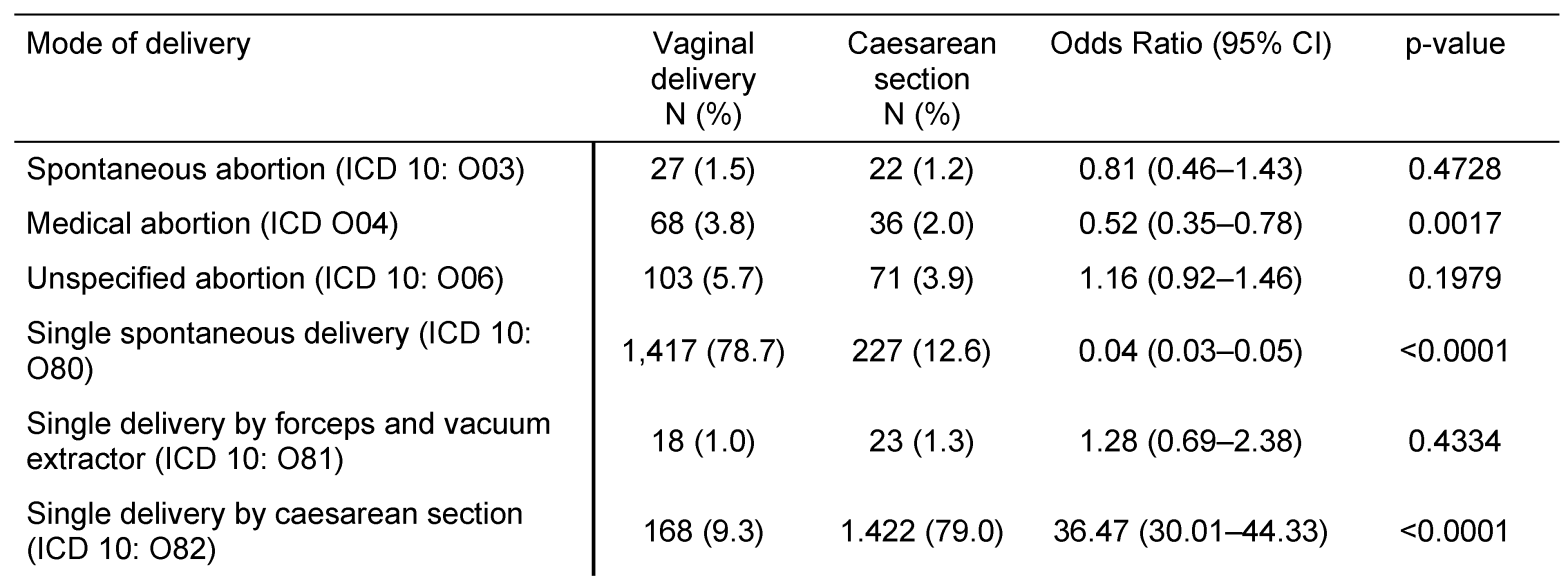
Mode of delivery in women with prior vaginal delivery vs. caesarean section in German gynecological practices

**Table 2:**
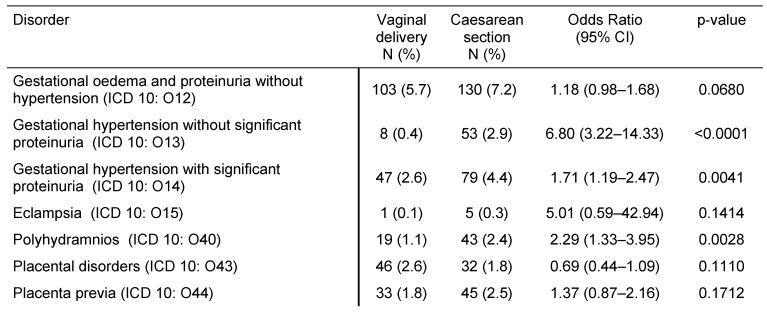
Pregnancy-induced maternal disorders in women with prior vaginal delivery vs. caesarean section in German gynecological practices

**Table 3:**
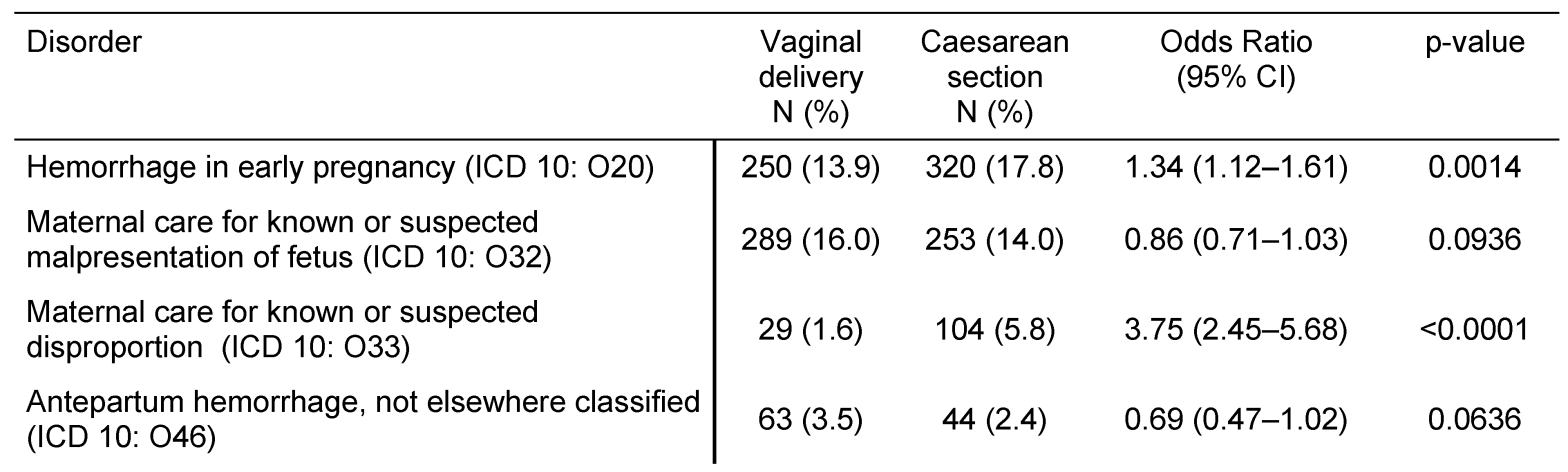
Pregnancy-associated diseases in women with prior vaginal delivery vs. caesarean section in German gynecological practices

**Table 4:**
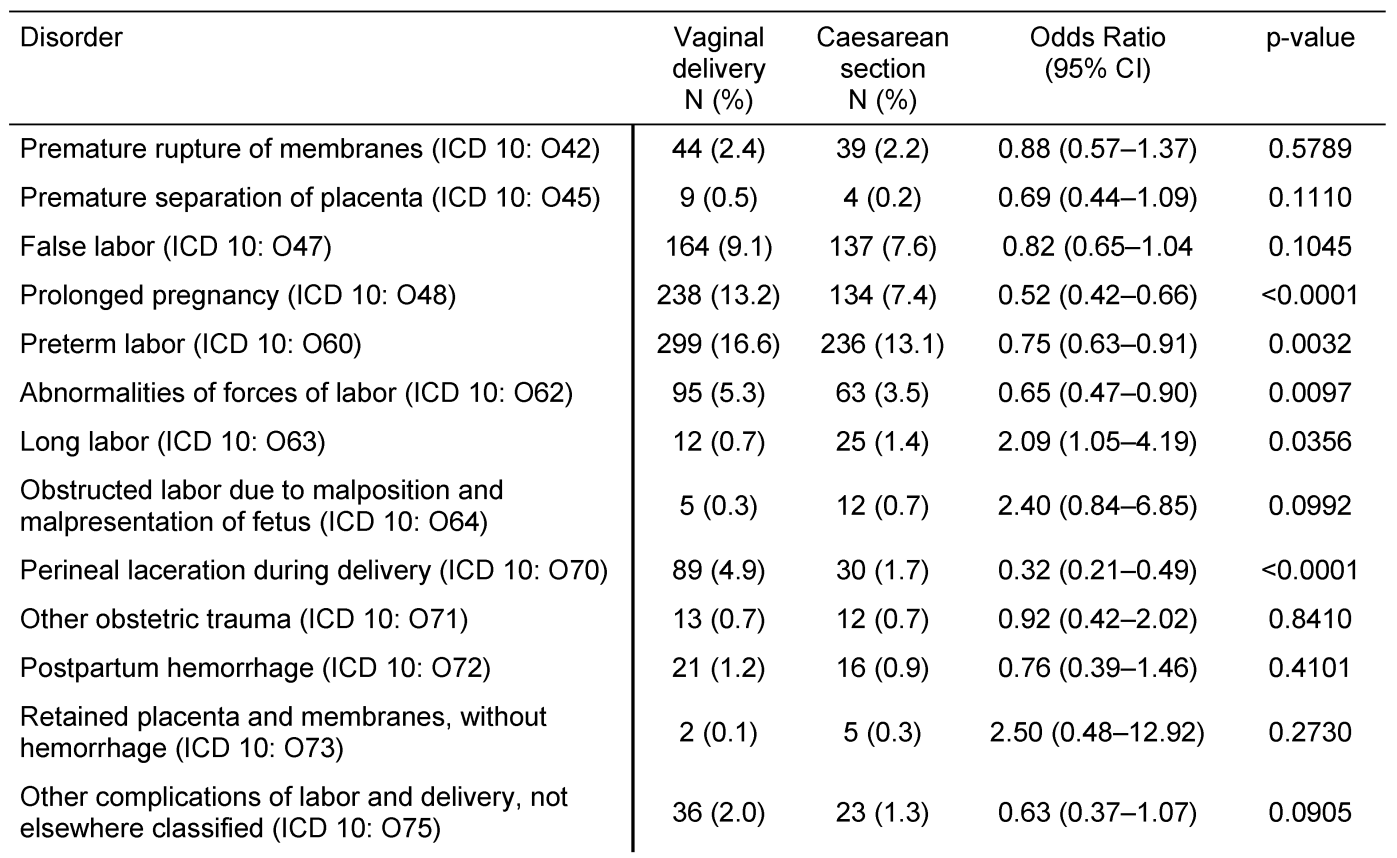
Complications prior to and during the birth in women with prior vaginal delivery vs. caesarean section in German gynecological practices

## References

[1] Black C, Kaye JA, Jick H (2005). Cesarean delivery in the United Kingdom: time trends in the general practice research database. Obstet Gynecol.

[2] Betrán AP, Merialdi M, Lauer JA, Bing-Shun W, Thomas J, Van Look P, Wagner M (2007). Rates of caesarean section: analysis of global, regional and national estimates. Paediatr Perinat Epidemiol.

[3] Stavrou EP, Ford JB, Shand AW, Morris JM, Roberts CL (2011). Epidemiology and trends for Caesarean section births in New South Wales, Australia: a population-based study. BMC Pregnancy Childbirth.

[4] Statistisches Bundesamt Mehr Krankenhausentbindungen 2014 bei gleicher Kaiserschnittrate. Pressemitteilung Nr. 338 vom 14.09.2015.

[5] Smith GC, Pell JP, Dobbie R (2003). Caesarean section and risk of unexplained stillbirth in subsequent pregnancy. Lancet.

[6] Silver RM, Landon MB, Rouse DJ, Leveno KJ, Spong CY, Thom EA, Moawad AH, Caritis SN, Harper M, Wapner RJ, Sorokin Y, Miodovnik M, Carpenter M, Peaceman AM, O'Sullivan MJ, Sibai B, Langer O, Thorp JM, Ramin SM, Mercer BM, National Institute of Child Health and Human Development Maternal-Fetal Medicine Units Network (2006). Maternal morbidity associated with multiple repeat cesarean deliveries. Obstet Gynecol.

[7] Bonifacio E, Warncke K, Winkler C, Wallner M, Ziegler AG (2011). Cesarean section and interferon-induced helicase gene polymorphisms combine to increase childhood type 1 diabetes risk. Diabetes.

[8] Curran EA, Dalman C, Kearney PM, Kenny LC, Cryan JF, Dinan TG, Khashan AS (2015). Association Between Obstetric Mode of Delivery and Autism Spectrum Disorder: A Population-Based Sibling Design Study. JAMA Psychiatry.

[9] Tollånes MC, Moster D, Daltveit AK, Irgens LM (2008). Cesarean section and risk of severe childhood asthma: a population-based cohort study. J Pediatr.

[10] Li HT, Zhou YB, Liu JM (2013). The impact of cesarean section on offspring overweight and obesity: a systematic review and meta-analysis. Int J Obes (Lond).

[11] Gurol-Urganci I, Cromwell DA, Mahmood TA, van der Meulen JH, Templeton A (2014). A population-based cohort study of the effect of Caesarean section on subsequent fertility. Hum Reprod.

[12] Becher H, Kostev K, Schröder-Bernhardi D (2009). Validity and representativeness of the "Disease Analyzer" patient database for use in pharmacoepidemiological and pharmacoeconomic studies. Int J Clin Pharmacol Ther.

[13] Jacob L, Taskan S, Sechet I, Ziller V, Kostev K (2016). Data from: Impact of caesarean section on mode of delivery, pregnancy-induced and pregnancy-associated disorders, and complications in the subsequent pregnancy in Germany.

[14] Lynch CM, Kearney R, Turner MJ (2003). Maternal morbidity after elective repeat caesarean section after two or more previous procedures. Eur J Obstet Gynecol Reprod Biol.

[15] Makoha FW, Felimban HM, Fathuddien MA, Roomi F, Ghabra T (2004). Multiple cesarean section morbidity. Int J Gynaecol Obstet.

[16] Guise JM, Eden K, Emeis C, Denman MA, Marshall N, Fu RR, Janik R, Nygren P, Walker M, McDonagh M (2010). Vaginal birth after cesarean: new insights. Evid Rep Technol Assess (Full Rep).

[17] Dodd JM, Crowther CA, Grivell RM, Deussen AR (2014). Elective repeat caesarean section versus induction of labour for women with a previous caesarean birth. Cochrane Database Syst Rev.

[18] Kyvernitakis I, Reichelt J, Kyvernitakis A, Misselwitz B, Hadji P, Schmidt S, Kalder M (2014). Trends of vaginal birth after cesarean delivery in Germany from 1990 to 2012: a population-based study. Z Geburtshilfe Neonatol.

[19] Taschner U, Schneck K (2012). Meine Wunschgeburt – Selbstbestimmt gebären nach Kaiserschnitt: Begleitbuch für Schwangere, ihre Partner und geburtshilfliche Fachpersonen.

[20] Snooks SJ, Swash M, Mathers SE, Henry MM (1990). Effect of vaginal delivery on the pelvic floor: a 5-year follow-up. Br J Surg.

[21] Rortveit G, Daltveit AK, Hannestad YS, Hunskaar S (2003). Urinary incontinence after vaginal delivery or cesarean section. N Engl J Med.

